# Identification and validation of novel engineered AAV capsid variants targeting human glia

**DOI:** 10.3389/fnins.2024.1435212

**Published:** 2024-08-13

**Authors:** Jessica Giacomoni, Malin Åkerblom, Mette Habekost, Alessandro Fiorenzano, Janko Kajtez, Marcus Davidsson, Malin Parmar, Tomas Björklund

**Affiliations:** ^1^Developmental and Regenerative Neurobiology, Lund Stem Cell Center, Department of Experimental Medical Science, Faculty of Medicine, Lund University, Lund, Sweden; ^2^Molecular Neuromodulation, Department of Experimental Medical Science, Faculty of Medicine, Lund University, Lund, Sweden

**Keywords:** AAV engineering, hGPCs, 3D culture, *ex vivo* brain slices, neuroscience, BRAVE library

## Abstract

Direct neural conversion of endogenous non-neuronal cells, such as resident glia, into therapeutic neurons has emerged as a promising strategy for brain repair, aiming to restore lost or damaged neurons. Proof-of-concept has been obtained from animal studies, yet these models do not efficiently recapitulate the complexity of the human brain, and further refinement is necessary before clinical translation becomes viable. One important aspect is the need to achieve efficient and precise targeting of human glial cells using non-integrating viral vectors that exhibit a high degree of cell type specificity. While various naturally occurring or engineered adeno-associated virus (AAV) serotypes have been utilized to transduce glia, efficient targeting of human glial cell types remains an unsolved challenge. In this study, we employ AAV capsid library engineering to find AAV capsids that selectively target human glia *in vitro* and *in vivo*. We have identified two families of AAV capsids that induce efficient targeting of human glia both in glial spheroids and after glial progenitor cell transplantation into the rat forebrain. Furthermore, we show the robustness of this targeting by transferring the capsid peptide from the parent AAV2 serotype onto the AAV9 serotype, which facilitates future scalability for the larger human brain.

## 1 Introduction

Recent research has demonstrated the ability of resident rodent glial cells, such as astrocytes and glial progenitor cells (GPCs), to convert into neurons via direct *in vivo* reprogramming (Liu et al., 2015; Torper et al., 2015; Pereira et al., 2017; Mattugini et al., 2019; Chen et al., 2020; Qian et al., 2020; Zhou et al., 2020). The conversion is initiated by overexpression or knockdown of key cell fate switches, such as transcription factors, regulatory proteins, or RNAs, which effectively reprogram the gene expression program of the glial cell into a neuronal one (Matsuda et al., 2019; Qian et al., 2020; Herrero-Navarro et al., 2021). Recombinant adeno-associated virus (AAV)-mediated delivery of the reprogramming factors is the preferred option for the clinical translation of this approach, but due to the lack of selective AAVs targeting glia, most experimental studies have used the glio- and neurotropic AAV2/AAV5 serotypes (Petrosyan et al., 2014; Torper et al., 2015; Qian et al., 2020). To restrict gene expression to glia, researchers have exploited glia-specific gene promoters (e.g., *GFAP*, *GfapABC(1)D*, *Gfa2*, *Aldh1l1*, and *NG2*) or rodent lines expressing Cre-recombinase driven by similar promoters to be used with recombination-dependent vectors (Lee et al., 2008; Torper et al., 2015; Qian et al., 2020). However, recent observations using stringent lineage-tracing strategies indicate that these systems fail to restrict gene expression to glia, leading to reporter labeling of endogenous neurons that are misinterpreted as reprogrammed neurons (Wang et al., 2021). This highlights the pressing need to develop AAVs with improved selectivity to overcome these challenges in glia-to-neuron reprogramming studies.

AAV has emerged as the preferred vector system for gene therapy, mainly due to its nonpathogenicity and ability to mediate long-term transgene expression in dividing and non-dividing cells without integration into the host genome (Podsakoff et al., 1994). AAV gene therapy has been approved for a number of diseases, including neurological disorders, and several advanced-stage clinical trials are ongoing (Issa et al., 2023; Lundstrom, 2023). However, the success of AAV-based therapeutics is limited by factors such as non-specific tropism of AAV vectors, pre-existing immunity, and inefficient large-scale production (Zincarelli et al., 2008; Ayuso, 2016; Fitzpatrick et al., 2018). Almost all AAV-based therapies that have reached clinical use rely on naturally occurring serotypes with evolutionary-honed properties that pre-define their characteristics (Kuzmin et al., 2021). While most AAV serotypes have evolved for broad cell-type transduction, which is crucial for viral replication and spread, this poses challenges for selective targeting of specific cell types as is often required for therapeutic applications. In the central nervous system (CNS), broad AAV tropism can partially be circumvented by injecting viral vectors directly into the brain area of interest. Post-transduction strategies, including the utilization of specific promoter/enhancer elements (Chan et al., 2017), cell-specific Cre-lox selection (Fenno et al., 2014), or transactivator-based approaches (Chen et al., 2022), as well as the incorporation of microRNA target sites in the untranslated regions of the viral transcript, can be used to achieve cell type specific targeting within a defined region (Akerblom et al., 2013; Keaveney et al., 2018). However, the transduction efficiency will inevitably limit the expression in the target cell type. Extensive off-target transduction increases dose requirements, raising the risk of immunological responses (Mingozzi and High, 2013) and scaling up costs for human applications (The editorial board, 2021). An alternative approach to achieve efficient targeting of a specific cell type with low off-target expression is to engineer the AAV capsid by displaying a peptide sequence at its surface. This modification alters the initial AAV tropism, and an extensive AAV capsid screening holds promise for discovering new capsid variants with the desired tropism.

The most common approach to AAV capsid engineering is directed evolution. Here, random processes such as capsid gene shuffling between known AAV serotypes (Grimm et al., 2008; Li et al., 2008; Asuri et al., 2012), insertion of degenerate sequences onto permissive sites of the production plasmid (leading to random amino acids; Girod et al., 1999; Rabinowitz et al., 1999; Wu et al., 2000; Maheshri et al., 2006; Dalkara et al., 2013; Korbelin et al., 2016), or phage display (Nicklin et al., 2001; Chen et al., 2009) are used to generate high-diversity pools of putative novel capsid variants. Most of those capsid variants will be dysfunctional or fail to form an AAV capsid, but through multiple generations of selective pressure and enrichment, novel variants with the desired property can be identified (Deverman et al., 2016; Chan et al., 2017; Ojala et al., 2018). Due to the random and linear selection processes (one selection pressure at a time), the method provides little mechanistic insights into the molecular targets engaged and is inherently challenging to reproduce. Increasing knowledge of the AAV capsid structure and its target interaction opens up an alternative approach based on rational design where fewer capsid variants are generated *de novo* and systematically evaluated and refined to improve the desired property (Bowles et al., 2012). However, due to the laborious process of this method, only a few hundred variants can be assessed compared to thousands or millions of variants possible with directed evolution (Yang et al., 1998; Grifman et al., 2001; Munch et al., 2013; Zinn et al., 2015; Kanaan et al., 2017; Tordo et al., 2018).

Moreover, novel capsid variants with improved features and functions identified through rational design or directed evolution have seldom transferred from the screening host species (predominantly mice) to humans or nonhuman primates (Hordeaux et al., 2018). This limitation largely confines their application to experimental studies. We have recently developed a third approach for capsid engineering named Barcoded Rational AAV Vector Evolution (BRAVE), which combines the broad screening diversity of directed evolution with the accuracy and reproducibility of rational design (Davidsson et al., 2019). The BRAVE approach builds on *in silico* modeling to generate a library of variants in the cell-binding domain of the AAV capsid. These variants are produced using oligonucleotide arrays on a scale of 10^5^ to 10^6^ and then cloned into the Cap gene of the AAV production plasmid. A degenerative DNA sequence (a.k.a. a molecular barcode) was inserted into the packaged genome’s untranslated region (UTR) to enable efficient readout of the capsid structure. A unique feature of the BRAVE approach is that the production plasmid library is stably replicated in bacteria in contrast to *in vitro* generation, where the peptides are inserted just before AAV production (e.g., in the CREATE approach; Deverman et al., 2016). This feature enables parallel screening on multiple tissues, species, and delivery routes to determine the optimal capsid variants for each target. Each screening experiment feeds data forward to the next, providing accurate information on species translatability, off-target transduction, and packaging efficacy. In this study, we used the BRAVE approach to engineer AAVs with improved transduction levels for human glia for experimental studies and clinical translation, focusing on their potential to enhance direct neural reprogramming approaches.

This study uses human embryonic stem cell (hESC)-derived GPCs *in vitro* and after transplantation into the adult rat brain to identify novel AAV capsid variants with glial tropism generated via the BRAVE approach. We identify and validate several novel AAVs with higher transduction efficiency and/or specificity for human glia.

## 2 Results

### 2.1 Brave library design and screening in glial spheroids

**Figure 1:**
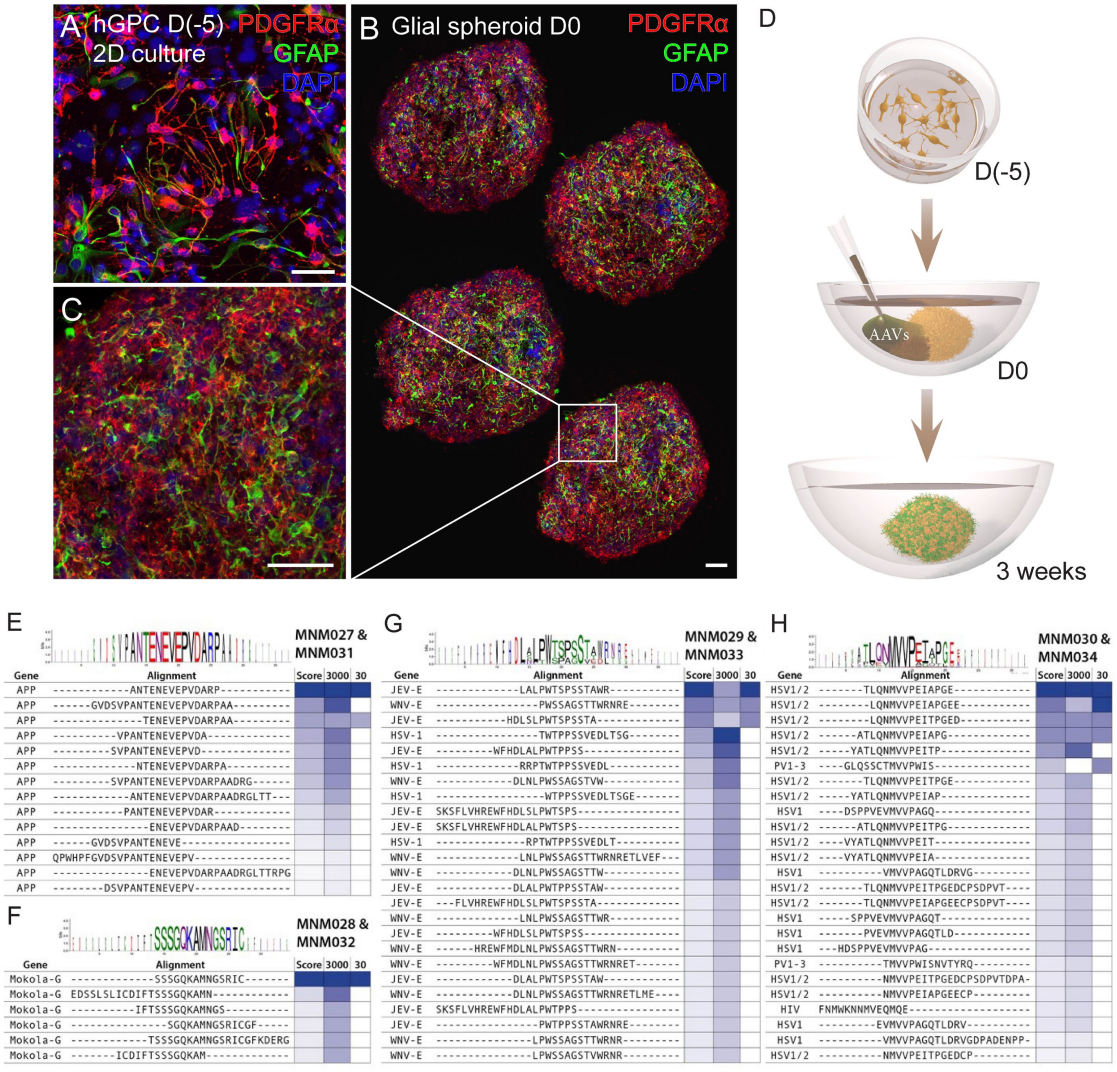
Workflow for glial spheroid formation and transduction with BRAVE library. **(A)** 2D culture of hGPCs expressing canonical glial markers (GFAP, PDGFRα) by immunocytochemistry. **(B,C)** Confocal immunofluorescence images of **(B)** four cryosections from a glial spheroid expressing glial markers on the day of AAV transduction **(B)**, with magnification **(C)**. **(D)** Schematic illustrating glial spheroid formation and viral transduction with corresponding time points. **(E–H)** Shared sequence homology and consensus motifs for the MNM027-MNM031 **(E)**, MNM028-MNM032 **(F)**, MNM029-MNM033 **(G)**, and MNM030-MNM034 **(H)** variants. Data information: 2D, two-dimensional; hGPCs, hESC-derived glial progenitor cells; D, day. Scale bars 50 μm.

We have previously established a protocol for generating hESC-derived GPCs *in vitro* and cryopreserving them at various time points (Nolbrant et al., 2020). Here, we used cell batches cryopreserved between days 207 and 347 of differentiation (Supplementary Table 1), containing a heterogenous population of highly proliferative hGPCs. At 2 weeks post-thawing, cell identity was confirmed by assessing the expression of the canonical cell-surface markers CD140 (PDGFRα) and CD44 using flow cytometry analysis and immunocytochemistry against PDGFRα and GFAP (Supplementary Table 1; Figure 1A). Hereafter, we cultured the hGPCs as spheroids to better recapitulate the complexity of cell–cell interaction, mimic the *in vivo* restrictions of vector diffusion, and overcome the viral dose-dependent cell death typically associated with monolayer cultures. To obtain glial spheroids, we seeded a single-cell suspension of hGPCs in low attachment 96-well plates, resulting in spheroid formation within 5 days. The glial spheroids maintained their expression of PDGFRα and GFAP, as confirmed by immunohistochemistry (Figures 1B,C) and were at this time point transduced with AAV2-based BRAVE libraries (Figure 1D).

The AAV capsid library applied here has previously resulted in the design of AAV capsid variants MNM001 to MNM026 with their own unique functions and transduction profiles (Davidsson et al., 2019). The plasmid library (which was used to produce the AAV library) was previously sequenced so that the capsid structure of each individual capsid variant could be linked to the mRNA-expressed molecular barcode. This look-up table (LUT) enables us to sequence the barcode from any transduced tissue and thereby derive the transduction profile of all 97,000 base variants and roughly 1,000,000 mutated variants within the library. These prior data were applied to select the most suitable capsid variants in the transduction of the glial spheroids, including efficient packaging, non-neuronal tropism, and absence of retrograde transport to connected brain regions but local transduction at the injection site.

Using this AAV capsid library, we performed two parallel transduction experiments in the glial spheroids. The first one with a high potency, lower precision library using 3,000 viral plasmid copies per cell at production (3,000 cpc), and the second with a high-fidelity, lower potency library produced with 30 plasmid copies per cell (30 cpc). The 3,000 cpc was expected to produce a mosaic AAV library, with each particle likely containing mosaic mutation capsid proteins and some cross-packaging of AAV genomes. The 30 cpc was assumed to make cells receive approximately one member from the AAV plasmid library (Maheshri et al., 2006) and subsequently generate a clean AAV library characterized by the same mutation capsid proteins and the corresponding barcode in each particle.

The spheroids were cultured for 3 weeks following transduction, and the AAV-derived GFP transcripts containing molecular barcodes were PCR-enriched from total cDNA, indexed, and deep sequenced. From the 30 cpc library transduction, we could identify successful infection of AAVs carrying 54,807 unique AAV-derived barcodes. In comparison, the 3,000 cpc library transduction resulted in the infection of AAVs carrying 127,004 unique barcodes. As quantifying the mRNA copies per barcode is highly sensitive to cell-type bias originating from the promoter expression, we implemented an alternative selection and de-noising approach based on barcode oversampling. The underlying principle of this approach lies in the utilization of over 3,500,000 distinct molecular barcodes to tag the 97,000 unique peptide variants. This means that, on average, nearly 50 barcodes are associated with the same peptide.

Consequently, if two AAV particles carry two unique barcodes, but both correspond to the expression of the same peptide within the AAV capsid, a transduction score of 2 is assigned. Our most potently transducing peptide was represented by 27 unique barcodes in the 3,000 cpc library and 3 barcodes in the 30 cpc library. This capsid variant was named MNM027. When we performed sequence similarity modeling in the screening dataset using the Hammock hidden Markov model (Krejci et al., 2016), we found that this peptide originates from the APP gene peptides partially overlapping with the “ENEVE” central amino acid sequence conferring similar tropism (Figure 1E). The next most abundant peptide in the 30 cpc library originated from the Mokola virus and was named MNM028 (Figure 1F). The next most abundant capsid variant in the 3,000 cpc originated from the Japanese encephalitis virus (JEV) with a central motif “LPWxSxSTW” shared with the West Nile virus (WNV) from the same Flaviviridae family (Figure 1G). This domain also shared sequence and functional homology with the unrelated Herpes Simplex virus (HSV), and the resulting capsid variant was named MNM029. Finally, the third most represented peptide in the 3,000 cpc library (represented by 20 unique barcodes) was also the top variant in the 30 cpc, with the peptide originating from HSV with a central “MVVPxI” motif also shared with the Poliovirus (PV; Figure 1H). This capsid was named MNM030.

The peptide insertion screening was conducted in the AAV2 capsid with the peptide inserted between amino acids 587 and 588, where wild-type (WT) AAV2 binds to heparan sulfate proteoglycans (HSPGs). While the AAV2 capsid is an excellent capsid for *in vitro* transduction, it falls short in producing high titers and does not have an attractive *in vivo* transduction pattern as AAV9, which also can be transported over the blood–brain barrier from systemic delivery. It was recently shown that a peptide named AAV2-retro (Tervo et al., 2016), designed for insertion into the same site of AAV2, could be transferred into the identical location of AAV9 with retained function (Lin et al., 2020). We, therefore, also moved the peptides from MNM027-MNM030 to the AAV9 capsid, and the corresponding capsid variants were named MNM031-MNM034. The novel peptide sequence was replacing amino acid Q588 in the AAV9 capsid gene, thus, it was inserted between amino acids 587 and 589.

### 2.2 *In vitro* validation of capsid variants targeting hGPCs

**Figure 2:**
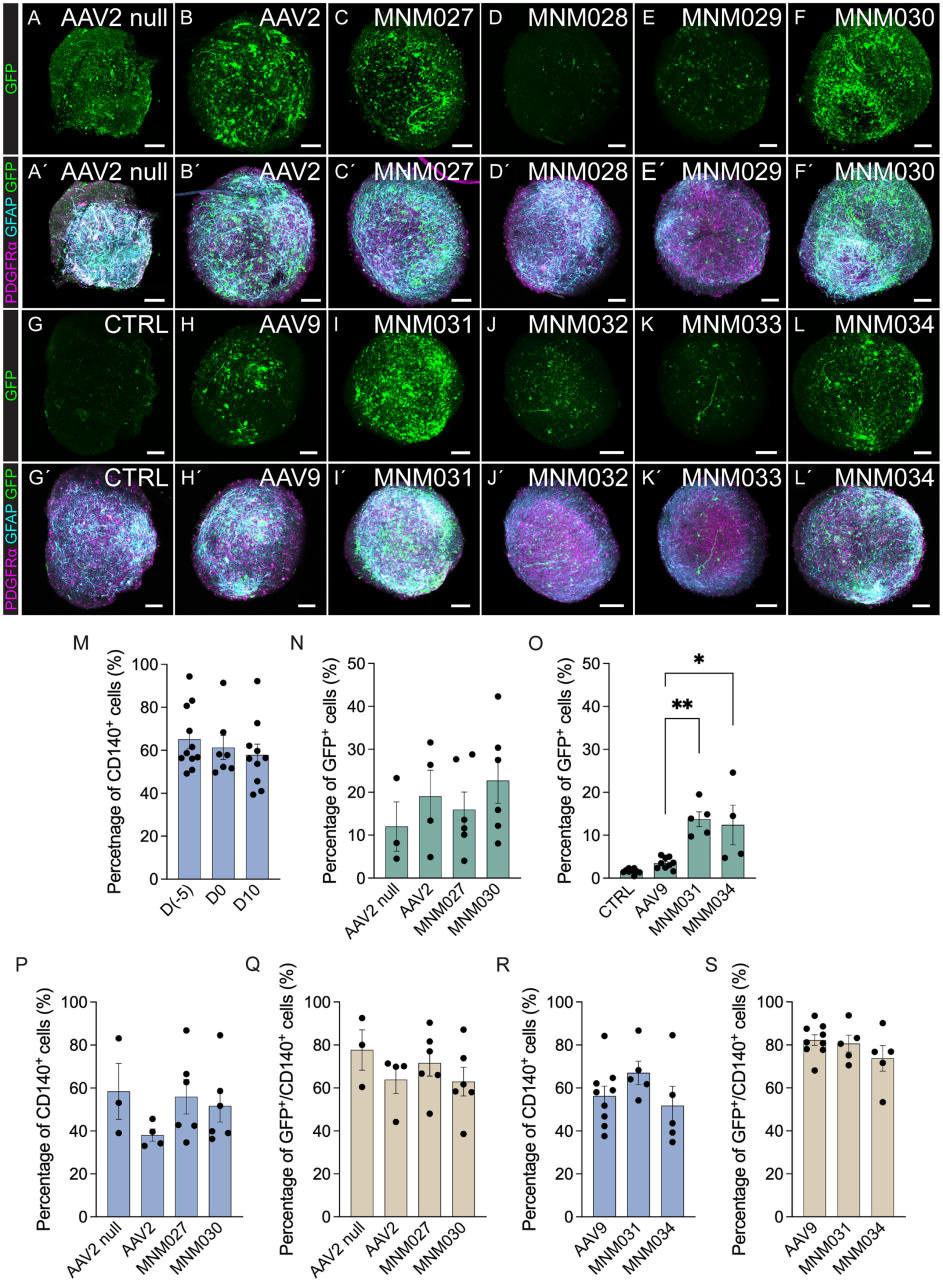
*In vitro* validation of AAV capsid variants at D10 post-transduction. **(AA´–LL´)** Representative immunofluorescence pictures of optically cleared whole glial spheroids expressing GFP **(A–L)** and glial markers (PDGFRα, GFAP) together with GFP **(A´–L´)** after transduction using *de novo* identified capsid variants **(CC´–FF,´II´–LL´)** and respective wild-type AAVs **(BB´,HH´)** compared to untransduced control **(GG´)** and AAV2 null **(AA´)**. The images represent maximum intensity projections. **(M)** Flow cytometry-based quantifications indicating the proportion of total CD140^+^ within glial cells at D(−5), glial spheroids at D0 prior to transduction, and untransduced glial spheroids at D10 of analysis. **(N–S)** Flow cytometry-based quantifications indicating the percentage of total GFP^+^ **(N,O)**, CD140^+^ **(P,R)**, and CD140^+^/GFP^+^ cells **(Q,S)** within glial spheroids transduced using novel capsid variants and parental serotypes. Data information: data are presented as means ± SEM, and all data points have been visualized in the graphs. Each data point represents a biological replicate (D(−5) *n* = 11; D0 *n* = 7; D10 *n* = 10; AAV2 null *n* = 3; AAV2 *n* = 4; MNM027 *n* = 6; MNM030 *n* = 6; AAV9 *n* = 9; MNM031 *n* = 5; MNM034 *n* = 4–5). One-way ANOVA non-parametric Kruskal-Wallis tests and follow-up multiple comparisons with uncorrected Dunn’s test are reported for all quantifications. ∗*p* ≤ 0.05 and ∗∗*p* ≤ 0.01 are shown; *p* > 0.05 is not shown. In **(O)** exact *p* = 0.0002, *p* = 0.0021 for AAV9 versus MNM031, *p* = 0.0153 for AAV9 versus MNM034. In (**N,O)**, AAV2 null and CTRL are excluded from statistical analysis. Scale bars 100 μm.

The *de novo* designed capsid variants and their respective parental serotypes were initially tested in glial spheroids to determine their effective glial tropism. At 10 days after transduction with 5×10^9^ viral particles per spheroid, the vector-derived GFP expression was amplified using immunofluorescence (Figures 2AA’–LL’) and quantified by flow cytometry (Figures 2N,O). Control samples of untransduced spheroids at D10 showed that the total proportion of cells positive for CD140 (PDGFRα) did not significantly vary (57.9 ± 5.0%; Figure 2M) when compared to the glial spheroids at D0 (61.3 ± 5.5%; Figure 2M) and cells at D(−5)(65.2 ± 4.5%; Figure 2M). AAV2 null and AAV2 transduced cells served as our references (Figures 2A,B,A’,B’), yielding a percentage of GFP^+^ cells (12.0 ± 5.7% and 19.8 ± 6.1% respectively; Figure 2N). Both MNM027 (15.97 ± 4.1%; Figures 2CC’) and MNM030 (22.73 ± 5.3%; Figures 2FF’) reintroduced the transduction ability at slightly higher efficiency (Figure 2N) while MNM028 (Figures 2DD’) and MNM029 (Figures 2EE’) resulted in minimal GFP expression. Interestingly, the MNM031 (Figures 2II’) and MNM034 (Figures 2LL’) variants (13.7 ± 1.7% and 12.4 ± 4.6% cells expressing GFP, Figure 2O), derived from the MNM027 and MNM030 variants, respectively, but inserted in the AAV9 capsid, retained the ability to transduce the spheroids with 4-fold higher efficiency than their WT serotype AAV9 (3.5 ± 0.4% GFP^+^; Figures 2HH’,O). GFP expression after MNM032 (Figures 2JJ’) and MNM033 (Figures 2KK’) transduction was also observed, but at significantly lower levels than MNM031 and MNM034-mediated expression (Figures 2II’, LL’). Untransduced glial spheroids showed that *in vitro* antibodies did not produce any non-specific staining (Figures 2GG’), and no GFP-expressing cells were detected through flow cytometry analysis (Figure 2O). On average, 72.2 ± 5.6% of the GFP^+^ cells per each capsid variant were found co-expressing CD140, indicating the ability of the designed capsid variants to efficiently target the most common glial subtype (Figures 2P–S; Supplementary Table 2).

### 2.3 Validation of capsid variants in *ex vivo* brain slice cultures

**Figure 3:**
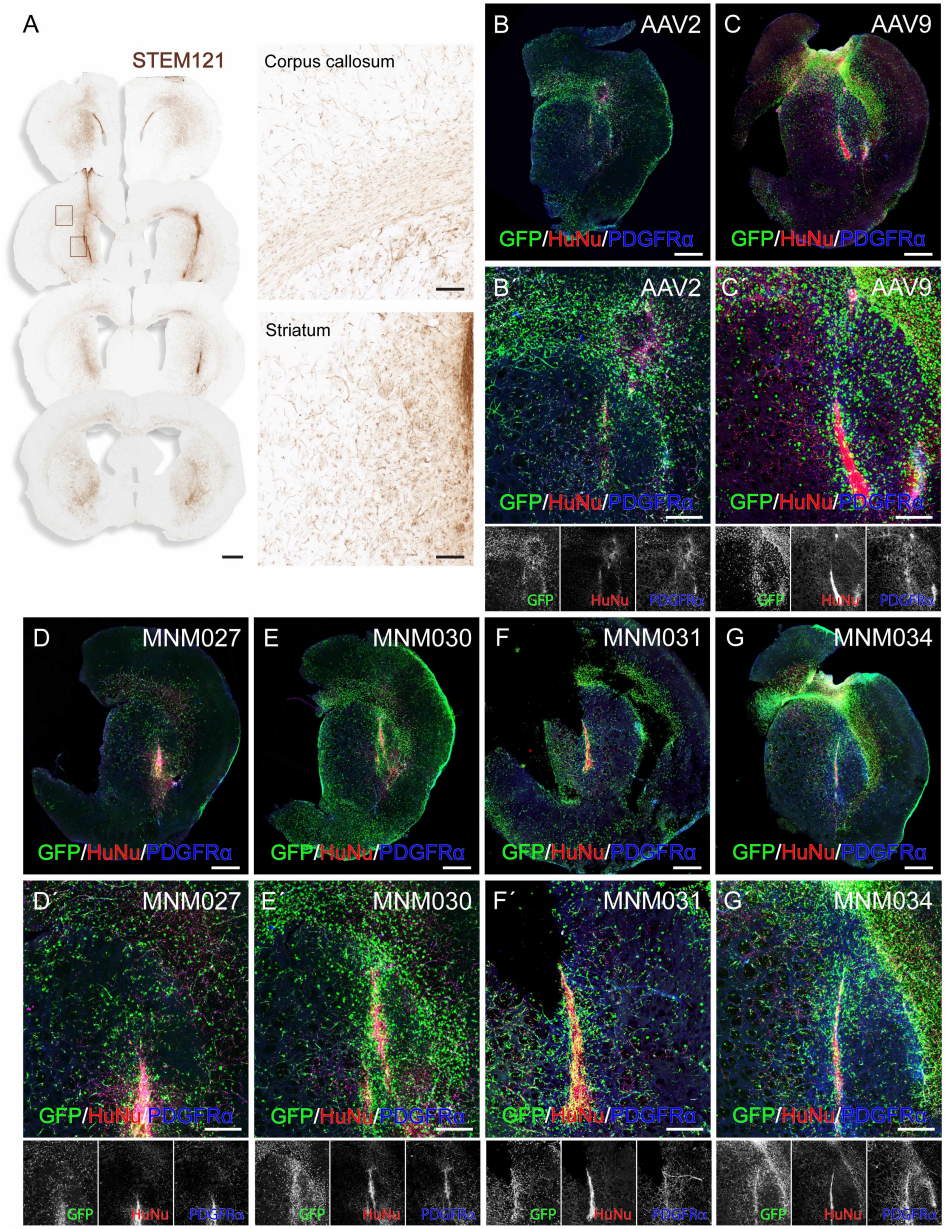
*Ex vivo* validation of capsid variants at D10 post-transduction. **(A)** DAB-developed STEM121 staining showing that hGPCs have migrated far beyond the graft core and dispersed as single cells 3 months after transplantation into the rat striatum, with illustrative magnifications. **(BB´–GG´)** Confocal immunofluorescence pictures of optically cleared whole brain slices exhibiting GFP expression after transduction using *de novo* identified capsid variants **(D–G)** and their respective wild-type AAVs **(B,C)**, with magnifications **(B´–G´)**. Human glial cells were identified using stainings for HuNu and PDGFRα. Maximum intensity projection of full 275 μm coronal slices. Data information: scale bars in **(A)** 1 mm (left) and 100 μm (right), in **(B–G)** 1 mm and in **(B´–G´)** 500 μm.

To exclude the possibility that differences in receptor expression between the *in vitro* and the *in vivo* environments alter the tropism of the selected variants and to evaluate their potential human specificity of transduction, we used a humanized xenograft rat model where the highly proliferative hGPCs were transplanted into the adult forebrain (Supplementary Table 3). At 3 months post-transplantation, the hGPCs had undergone substantial expansion in the host brain. They were dispersed extensively across a significant portion of the injected hemisphere, expanding to the cortex via the corpus callosum (Figure 3A). At this time point, we prepared acute brain slices of the transplanted region for *ex vivo* culturing. We transduced them on the following day with the *de novo* designed capsid variants and their respective parental serotypes. This method allows for the parallel investigation of different AAV serotypes by transducing different brain slices prepared from the same brain. On the day of transduction, the brain slices displayed canonical markers confirming the GPC identity of the human transplanted cells (Supplementary Figures 1A,B). 1.5 × 10^10^ viral particles per capsid variant were delivered by placing a viral suspension droplet onto the center of the slice. The tissue was then fixed and processed for analysis 10 days post-transduction (Supplementary Figure 1C). To systematically quantify human glia-specific and serotype-dependent AAV administrations, we applied a high-content screening approach combined with optical tissue clearing after whole brain slice staining for GFP, the canonical glial marker PDGFRα and the human marker HuNu (Figures 3B–G).

**Figure 4:**
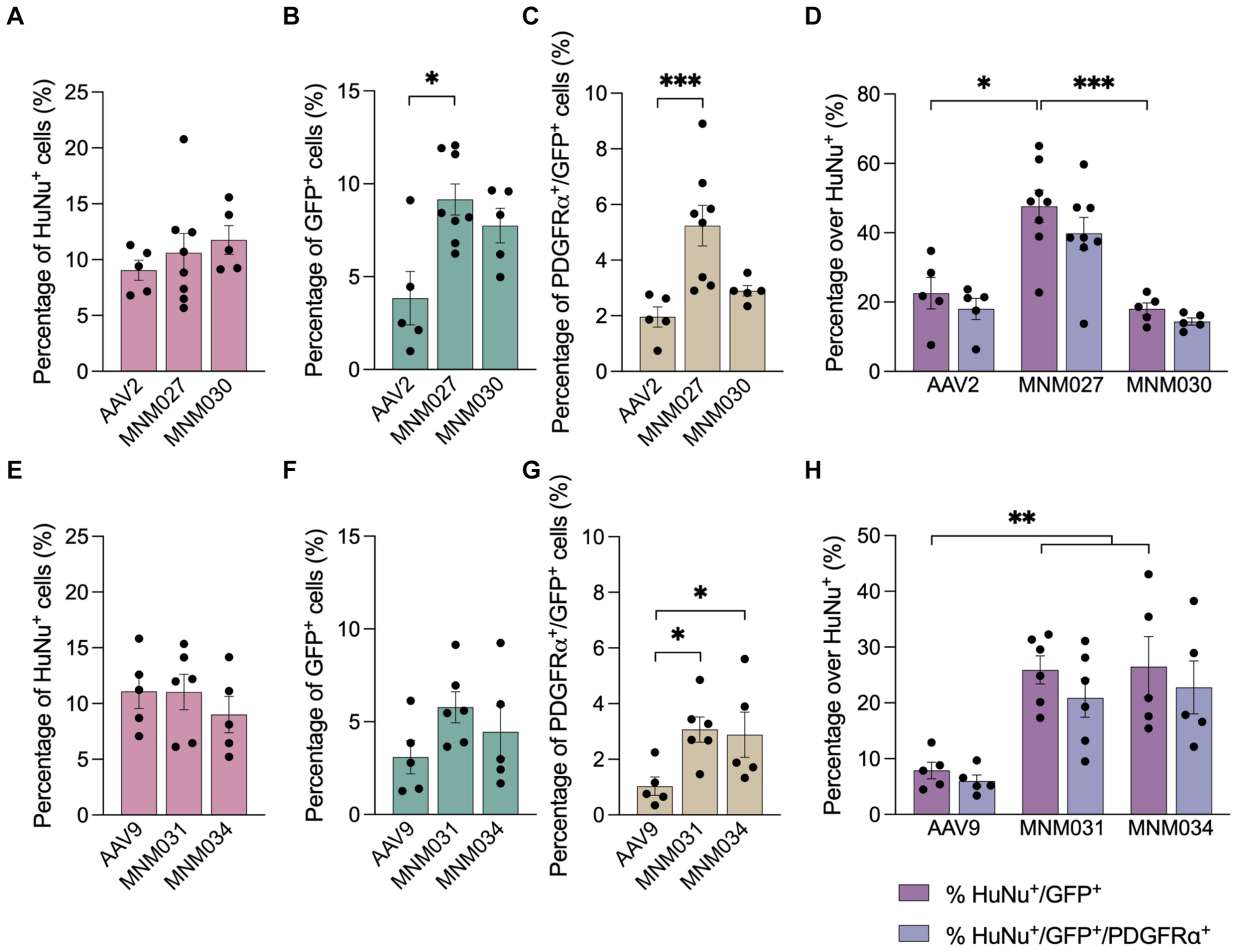
*Ex vivo* brain slice cultures offer improved predictive capability for assessing capsid variant specificity. **(A–G)** Operetta CLS-based quantifications showing the total percentage of HuNu^+^ hGPCs **(A,E)** GFP-expressing cells **(B,F)** and PDGFRα^+^/GFP^+^ cells **(C,G)** per viral condition. The analysis was restricted to brain regions where human cells accounted for 5% or more of the total DAPI^+^ cells per field. **(D,H)** Percentage of human cells co-expressing GFP and/or PDGFRα per capsid variant quantified with Operetta CLS. The analysis was restricted to brain regions where human cells accounted for 5% or more of the total DAPI^+^ cells per field. Data information: data are presented as means ± SEM, and all data points have been visualized in the graphs. Each data point represents a biological replicate (AAV2 *n* = 5; MNM027 *n* = 8; MNM030 *n* = 5; AAV9 *n* = 5; MNM031 *n* = 6; MNM034 *n* = 5). One-way ANOVA non-parametric Kruskal-Wallis tests and follow-up multiple comparisons with uncorrected Dunn’s test are reported for HuNu^+^, GFP^+^, PDGFRα^+^/GFP^+^ and HuNu^+^/GFP^+^ quantifications. ∗*p* ≤ 0.05, ∗∗*p* ≤ 0.01 and ∗∗∗ *p* ≤ 0.001 are shown; *p* > 0.05 is not shown. In **(B)** exact *p* = 0.0370, *p* = 0.0134 for AAV2 versus MNM027. In **(C)** exact *p* = 0.0001, *p* = 0.0004 for AAV2 versus MNM027. In **(D)** exact *p* = 0.0005, *p* = 0.0192 for AAV2 versus MNM027, *p* = 0.0017 for MNM027 versus MNM030. In **(G)** exact *p* = 0.0180, *p* = 0.106 for AAV9 versus MNM031 and *p* = 0.0395 for AAV9 versus MNM034. In **(H)** exact *p* = 0.0023, *p* = 0.0055 for AAV9 versus MNM031, *p* = 0.0079 for AAV9 versus MNM034. In **(D,H)**, the percentages of HuNu^+^/GFP^+^ cells were compared to the percentages of HuNu^+^/GFP^+^/PDGFRα^+^ cells for each viral condition using multiple Mann–Whitney tests (alfa = 0.05), which revealed no significant differences.

Using the Operetta CLS automated spinning disk confocal microscope, we imaged the entire brain sections (*n* = 5–8 per viral condition) and performed image analysis on selected brain regions with 5% or more HuNu^+^ cells out of total counted DAPI^+^ nuclei per field (Supplementary Figure 1C). This resulted in a similar total fraction of human cells (averages of 10.5 ± 0.9% for AAV2-derived variants and AAV2 WT as well as 10.4 ± 0.9 for AAV9-derived variants and AAV9 WT; Supplementary Table 4) per each analyzed brain section (Figures 4A,E). Next, we used these selected fields to assess the transduction efficiency at the experimental time point of analysis. Based on GFP expression, MNM027 (Figures 3DD’) targeted 9.2 ± 0.8% cells (Figure 4B), which was significantly higher than the 3.8 ± 1.4% AAV2-transduced cells (Figures 3BB’, 4B), followed by a similarly efficient performance for MNM030 (Figures 3EE’) which targeted 7.7 ± 0.9% cells (Figure 4B). MNM031 (Figures 3FF’) and MNM034 (Figures 3GG’) resulted in a similar number of GFP-expressing cells, 5.8 ± 0.8% and 4.5 ± 1.4% (Figure 4F), respectively, which was comparable to the 3.1 ± 0.9% GFP^+^ cells quantified for AAV9 (Figures 3CC’, 4F). Additionally, MNM027 also targeted a higher proportion of PDGFRα^+^ cells, 5.2 ± 0.7%, compared to the 1.9 ± 0.4% and 2.9 ± 0.2% of PDGFRα^+^/GFP^+^ cells transduced by AAV2 and MNM030, respectively (Figure 4C). Conversely, also MMN031 and MNM034 were more effective in targeting PDGFRα^+^ cell populations with 3.1 ± 0.5% and 2.9 ± 0.8% PDGFRα^+^/GFP^+^ transduced cells respectively, compared to the 1.0 ± 0.3% PDGFRα^+^/GFP^+^ cells transduced by AAV9 (Figure 4G). By limiting the GFP quantifications to the human counterpart, we were able to assess that 47.6 ± 4.7% of the MNM027 targeted cells co-localized with HuNu, while only 22.6 ± 4.5% and 18.0 ± 3.9% GFP^+^ were of human origin for AAV2- and MNM030-transduced cells, respectively (Figure 4D). These data show that MNM027 can transduce transplanted hGPCs more efficiently than MNM030. AAV9-mediated delivery resulted in a total of 7.9 ± 1.5% GFP^+^/HuNu^+^, which was significantly lower compared to both MNM031 and MNM034 with 25.9 ± 2.5% and 26.5 ± 5.4% cells co-localizing with HuNu, respectively (Figure 4H). This aligns with the 4-fold increased transduction efficiency previously observed for MNM031 and MNM034 *in vitro* using glial spheroids. Furthermore, the GFP^+^ human cells highly co-localized with PDGFRα for all analyzed conditions (Figures 4D,H; Supplementary Table 4). In accordance with the results obtained in spheroids, we did not detect any differences in selectivity for hGPCs between the variants MNM028 (Supplementary Figure 1DD´), MNM029 (Supplementary Figure 1EE´) and MNM032 (Supplementary Figure 1FF´), which showed little to no GFP expression.

## 3 Discussion

To advance direct *in vivo* reprogramming for clinical brain repair, it is crucial to generate novel variants of AAV capsids capable of efficiently targeting human glia *in vitro* and *in vivo.* Thus far, multiple efforts have been made to improve the glial tropism and specificity through capsid engineering. Using peptide insertion into the AAV9 capsid, Kunze et al. showed that the peptide RGDLGLS (AAV9P1) conferred improved transduction of astrocytes *in vitro* (Kunze et al., 2018). Unfortunately, when assessing this capsid variant *in vivo*, it displayed much higher skeletal muscle tropism than glial tropism and was renamed AAVMYO (Weinmann et al., 2020). Another attempt to generate gliotrophic AAV capsids was conducted by Deverman et al. using the CREATE approach, where a *GFAP*-Cre-driver transgenic mouse line is utilized to select for on-target transduction using directed evolution (Deverman et al., 2016). Among the several variants identified in this study, the most noteworthy is the php.B variant displaying tropism to mouse glia combined with high neurotropism *in vivo*.

Moreover, a notable challenge is its reliance on the Ly6A receptor for cell adhesion, which is only expressed in C57Bl6 mice and related strains, excluding its broader application in higher species, including humans (Huang et al., 2019). In the same study, a second capsid AAV-PHP.A was developed showing higher specificity for mouse glia *in vivo.* When tested on human iPSC-derived cortical spheroids *in vitro*, however, only approximately 15% of AAV-PHP.A transduced cells exhibited GFAP-positivity compared to 40% for AAV-PHP.B, suggesting a small Ly6A-independent uptake with an unknown mechanism. Hence, there is a critical need for novel screening models that enable direct assessment in predictive human cell systems.

The hESC-derived GPCs resemble *bona fide* human GPCs and reprogram *in vitro* with comparable timing and efficiencies into subtype-specific neurons (Nolbrant et al., 2020), making them an essential model for investigating and developing direct reprogramming of human glia into therapeutic neurons. In this study, we used the hESC-derived GPC-model as a screening system for AAV engineering based on the BRAVE methodology (Davidsson et al., 2019) with the goal of developing better-suited AAV capsids for *in vivo* targeting of human glia to support experimental studies and future therapeutic applications. The initial screens were performed in a 3D culture system using both a high-titer, lower-precision library and a high-fidelity, lower-titer library. These screens identified four capsid variants (MNM027-MNM030) with high targeting efficiency to human glia *in vitro*. The designed peptides were also transferred to the AAV9 capsid (MNM031-MNM034) because of its unique ability to transduce the CNS following intravenous injection, which can be advantageous in future therapeutic development. Although glial spheroids serve as a valuable initial screening system, they may not entirely replicate targeting *in vivo*, where receptors involved in viral transduction often differ from those observed *in vitro*. This can be due to complex tissue interactions, varying microenvironmental conditions, developmental states, species-specific differences and post-translational modifications. Glial spheroids cannot be used to assess specificity for transducing human cells and predict translational outcomes. Therefore, we also tested the capsid variants in a humanized xenograft model established by the transplantation of hGPCs to the adult rat brain. At 3 months post-grafting, when the hGPCs have expanded significantly in the host brain, brain slice cultures were established and subsequently transduced with engineered and WT AAV capsid variants. The results showed a better maintained *in vivo* transduction potency of the MNM AAVs compared to the WTs. Furthermore, our findings suggest potential species preference with MNM027 selectively transducing hGPCs compared to MNM30, which, despite a comparable percentage of GFP-expressing cells, targets hGPCs with lower efficiency.

This study shows that the combination of prior packaging and transduction data with single glial spheroids can, thanks to the parallel nature of the approach, be sufficient to identify functional capsids with the desired on-target transduction. This unique property of the BRAVE technology hinges on several key components. Firstly, the barcode oversampling and high diversity library essentially rendered the single spheroid into over 50,000 parallel transduction experiments. Secondly, the reusable plasmid library allows for the incorporation of all prior packaging and transduction data, *in vitro* and *in vivo,* into our computer modeling pipeline to identify only those capsid variants with a proven capacity for efficient packaging. At the same time, we can filter out the variants that are transducing other cell types as well, e.g., rat neurons. Of note is, however, that the computational prediction is not perfect. While the MNM027, MNM030, MNM031, and MNM034 performed as expected, the transduction efficacy of MNM028, MNM029, MNM032, and MNM033 did not perform as predicted by the computational modeling. The cause of this discrepancy needs to be fully elucidated, but a possible explanation is that the peptide in the MNM028/MNM032 variants differed through two substitutions from the synthesized peptide on the gene chip array. That is significantly more than expected and may be caused by sequencing errors in the LUT generation. It could then not reflect actual substitutions in the peptide whereby we produced the wrong peptide variant for validation. The underperformance of the MNM029/MNM033 variants may have a distinct underlying cause. In our BRAVE library, peptides are inserted with linkers of varying lengths and rigidity, including a single alanine as well as a flexible and a rigid 5 amino acid linker (see Davidsson et al., 2019 for details). In the glial spheroid screening, only 2 out of 23 barcodes reflected the single alanine linker, while the remaining 21 were divided between the two 5 amino acid linkers. Nonetheless, we opted for the single alanine linker to maintain consistency in the capsid structure with the other variants, which may have influenced the outcome.

While MNM027 efficiently transduced the glial spheroids *in vitro*, the difference was not statistically significant compared to the WT variant. At first glance, this might seem surprising, given that it outperformed the WT variant in the brain slice cultures, which was evident in both the percentage of total transduced cells and the percentage of targeted human cells co-expressing GFP^+^. However, the AAV2 WT serotype relies on the abundant HSPGs for cell adhesion, favoring *in vitro* transduction. This transduction superiority does not translate to *in vivo* settings, where AAV2 is one of the less efficient WT AAV serotypes in the CNS. The MNM variants have this moiety removed to reduce ubiquitous transduction and would, therefore, transduce very inefficiently without the insertion of retargeting peptides (Davidsson et al., 2019). Instead, we observe a switch in cell adhesion from HSPGs to other yet-to-be-determined receptors. However, the similar performance of AAV2 null, which lacks HSP binding (through p.N587_R588insAS insertion), suggests a potential additive or competitive role in the *in vitro* uptake mechanism of a different surface domain that does not originate from the *de novo* designed peptides. On the other contrary, MNM031 and MNM034 demonstrated consistent superiority both *in vitro* and *ex vivo*, exhibiting a higher total transduction efficiency and a greater degree of human glia selectivity compared to the AAV9 WT serotype, which lacks the binding site favoring the AAV2-derived serotypes.

In summary, the findings in this study show that the transduction potency of the BRAVE-designed AAVs as assessed *in vitro* is also preserved *ex vivo,* resulting in the increase of both transduction efficiency (for MNM027) and specificity (for MNM027, MNM031, and MNM034) as observed through the *ex vivo* brain slice cultures. Going forward, these results provide a powerful basis for *in vivo* applications using humanized rodents, including AAV-mediated direct conversion, where the novel highly transducing and selective capsid structures could improve the delivery of neural reprogramming factors to human glia.

## 4 Materials and methods

### 4.1 BRAVE AAV library generation

The AAV plasmid library was generated as described previously (Davidsson et al., 2019). The backbone plasmid used for cloning of the barcoded modified AAV capsids was developed from a self-complementary AAV vector expressing GFP (pscAAV-GFP; Gray and Zolotukhin, 2011) together with components from pDG (Grimm et al., 1998; Adenovirus genes VA, E2A, and E4 are deleted). The final plasmid contained an eGFP expression cassette driven by a CMV promoter and the wild-type AAV2 Rep/Cap genes controlled by the mouse mammary tumor virus (MMTV) promoter. A NheI site was introduced between sequences of N587 and R588 of VP1 capsid protein for peptide insertion.

### 4.2 Selecting proteins for peptide display

The library constructed in Davidsson et al. (2019) was repurposed for this screening. Briefly, 131 proteins were selected, belonging to five categories: neurotropic viruses, lectins, neurotrophins, neurotoxins, and neuronal proteins. Peptides were designed to be incorporated between N587 and R588 of VP1 capsid protein (Girod et al., 1999). This site was previously reported to tolerate the insertion of large peptides (Ried et al., 2002; Michelfelder and Trepel, 2009) and block heparan sulfate proteoglycan binding (Opie et al., 2003; Perabo et al., 2006). Four different peptide conformations were designed as A-14aa-A, A-22aa-A, A5-14aa-A5, G4S-14aa-G4S. They contained two lengths of peptides of 14 or 22 amino acid (aa) residues and were flanked by either a spacer of one amino acid of alanine (A) or a short linker (A5 or G4S; Chen et al., 2013; Reddy Chichili et al., 2013).

All possible unique peptides of 14 or 22aa from the candidate proteins were identified and generated by a sliding window approach using a purpose-built R workflow (see Data Availability Statement). The final oligonucleotide pool containing 92,918 unique oligonucleotides was synthesized using a 90,000 DNA array (Custom Array). Gibson assembly was used to insert the oligonucleotide pool (into the capsid gene located outside of the ITRs) and barcodes (downstream of GFP located inside of the ITRs) to generate a barcoded AAV plasmid library.

### 4.3 Production of AAV vector library

AAV production was performed as described previously (Negrini et al., 2020). Briefly, HEK293T cells were seeded in 175 cm^2^ cell culture flasks to achieve 60–80% confluency before transfection. 25 μg (3,000 cpc), 250 ng (30 cpc) or 25 ng (3 cpc) of the AAV plasmid library and 46 μg of pHGTI-adeno1 (Streck et al., 2005) were transfected using standard PEI transfection (Gray et al., 2011). PEI and plasmid DNA were mixed in 3 mL DMEM, incubated for 15 min, and then added to the cells. The molar ratio of AAV plasmid library: pHGTI-adeno1 were 1:1 (3,000 cpc) 0.01:1 (30 cpc), respectively. At 16 h post-transfection, 27 mL of medium was replaced with OptiPRO serum-free medium (Thermo Fischer Scientific). AAVs were harvested 72 h post-transfection using polyethylene glycol 8,000 precipitation and chloroform extraction followed by phosphate-buffered saline exchange in Amicon Ultra-0.5 Centrifugal filters (Merck Millipore). Purified AAVs were diluted 50-fold and treated with 2 units of DNase I (NEB) for 10 min at 37°C, followed by heat inactivation at 65°C for 10 min. The DNAse treated AAV was diluted 1:500,000 fold, and 2 or 4 mL of AAV was mixed with primers for ITR (CGGCCTCAGTGAGCGA and GGAACCCCTAGTGATGGAGTT, final concentration 40 nM) and 1x QX200^™^ ddPCR^™^ EvaGreen Supermix (Bio-Rad) in a final volume of 25 mL. Each reaction was then loaded into the sample well of an 8-well disposable cartridge (Bio-Rad) along with 70 μL of droplet generation oil (Bio-Rad), and droplets were formed in a droplet generator (Bio-Rad). Droplets were then transferred to a 96-well PCR plate, heat-sealed with foil, and amplified to the endpoint with a conventional thermal cycler (95°C for 10 min, followed by 39 cycles of 94°C for 30 s and 57°C for 1 min followed by a final 98°C heat treatment for 10 min). The PCR plate was subsequently scanned on a QX100 droplet reader (Bio-Rad), and the data were analyzed with QuantaSoft software (Bio-Rad). The following AAVs were produced: AAV2null-CMV.GFP, AAV2-CMV.GFP, MNM027-CMV.GFP, MNM028-CMV.GFP, MNM029-CMV.GFP, MNM030-CMV.GFP, AAV9-CMV.GFP, MNM031-CMV.GFP, MNM032-CMV.GFP, MNM032-CMV.GFP, MNM033-CMV.GFP and MNM034-CMV.GFP with titers ranging from 1.2 x 10^11^ to 2.8 x10^13^ genome copies per milliliter.

### 4.4 Sequencing AAV plasmid library

To facilitate paired-end Illumina sequencing of the plasmid library, a part of the AAV plasmid was excised by Cre-recombinase to bring the inserted peptide sequence and barcode closer together [DNA was amplified by emulsion PCR with P5/P7 Illumina adapter primers using Phusion HSII (Thermo Fischer Scientific)]. The emulsion PCR product was purified and size selected using SPRIselect Kit (Beckman Culter). The purified and indexed PCR products were sequenced using Illumina NextSeq Reagent Kit v2 (Illumina) with 150 bp paired-end sequencing.

### 4.5 hESC-derived GPC culture

Undifferentiated RC17 (Roslin Cells, cat.no. hPSCreg RCe021-A, p26–30) were expanded in IPS-Brew XF medium (StemMACS, Miltenyi) on LN521 (0.5 μg/cm^2^, Biolamina)-coated tissue plates at 37°C in 5% CO_2_ and passaged weekly with ethylenediaminetetraacetic acid (EDTA, 0.5 mM, Gibco, Thermo Fisher). hGPCs were derived from hESCs and cryopreserved as previously described (Nolbrant et al., 2020). Cryopreserved vials of hGPCs are thawed with the ThawSTAR Automated Cell Thawing Instrument and spun down for 5 min at 300xg in wash medium (DMEM with 5% KSR).

### 4.6 Generation of glial spheroids and *in vitro* viral transduction

hESC-derived GPCs were seeded onto poly-L-ornithine (100 μg/mL; Sigma-Aldrich) and laminin (5 μg/mL; Thermo Fisher) coated tissue culture plates (Corning) and kept in glial medium containing DMEM/F12 basal medium, B27 supplement, N1 supplement (Sigma-Aldrich), MEM NEAA, Antibiotic-Antimycotic, T3 (60 ng/mL; Sigma-Aldrich), db-cAMP (1 μM; Sigma-Aldrich), Biotin (100 ng/mL; Sigma-Aldrich), recombinant human PDGF-AA protein (10 ng/mL; R&D Systems), recombinant human IGF-I (10 ng/mL; R&D Systems) and recombinant human NT-3 Protein (10 ng/mL; R&D Systems). At 2 weeks post-thawing, hGPCs were mechanically detached from the plates using a cell scraper, dissociated at single cell level with Accutase (Thermo Fisher Scientific), and analyzed by flow cytometry for CD44 and CD140 and by immunocytochemistry for various glial markers. Glial spheroids were formed by self-aggregation of 100,000 single cells in a 96-well ultra-low attachment U-bottom plate (Corning) in glial medium within 5 days. At day 5 in culture, AAV transduction of glial spheroids was performed in 50 μL of glial medium at a multiplicity of infection (MOI) of 160,000 with the 3,000 cpc BRAVE library, 50,000 with the 30 cpc BRAVE library and 5,000 for each *de novo* generate capsid variants and parental serotypes. On the following day, 60 μL of glial medium was added to the cultures, and at 72 h post-transduction, the medium was replaced entirely.

### 4.7 Flow cytometry analysis

For quality control assessment of glial identity and quantification of GFP-expressing transduced cells, monolayer or spheroid hGPC cultures were dissociated using Accutase (Thermo Fisher Scientific) for 8 min. Subsequently, 100,000 cells were resuspended in 100 μL of Miltenyi wash buffer (containing PBS, 0.5% BSA Fraction V, 2 μM EDTA, and 0.05‰ Phenol Red). Flouorochrome-labeled antibodies (PE anti-human CD140a, BD Biosciences, cat. no. 556002, 1:10; APC anti-CD44, Miltenyi, cat. no. 130–095-177, 1:500; APC anti-human CD133/1, Miltenyi, cat. no. 130–113-668, 1:50; FITC anti-human SSEA-4, Biolegends, cat. no. 330410, 1:20) were added, and cells incubated for 15 min at 4°C. After washing in Miltenyi wash buffer for 10 min at 200xg, cells were transferred to 5 mL polystyrene tubes with cell-strainer caps in DMEM/F12 with DNAse. Propidium iodide (PI, Miltenyi, cat. no. 130–095-177, 1:500) was used to exclude dead cells from the analysis. Single live cells were analyzed on a FACSAria III sorter (BD Biosciences), gates were set based on Fluorescence Minus One controls and compensation performed using single-stained cells. Flow cytometry data were analyzed using the software FlowJo 10.8.

### 4.8 Transplantation of hGPCs into the rodent brain

All procedures were performed in accordance with the European Union Directive (2010/63/EU) and approved by the local ethical committee at Lund University, as well as the Swedish Department of Agriculture (Jordbruksverket). Female nude athymic rats (Hsd:RH-*Foxn*1^rnu^) were purchased from Envigo. All rats were housed in ventilated cages with *ad libitum* access to food and water under a 12 h-light and dark cycle. All surgeries were performed under general anesthesia via intraperitoneal injections of ketaminol (45 mg/kg) and dormitor (0.3 mg/kg). Animals were placed into a stereotaxic frame, and the tooth bar was adjusted to the flathead position. Cells were transplanted either unilaterally or bilaterally to the striatum of adult rats older than 16 weeks. For unilateral transplants (*n* = 2), we injected 300,000 cells in 6 μL of HBSS over four 1.5 μL deposits (50,000 cells/μl) to the striatum (AP +0.9, ML −3.0, DV −4.5/−5.5 and AP +1.4, ML −2.6, DV −4.5/−5.5). For bilateral transplants (*n* = 5), we injected 900,000 cells in 12 μL of HBSS over eight 1.5 μL deposits (75,000 cells/μl) to the striatum (AP +0.9, ML ±3.0, DV −4.5/−5.5 and AP +1.4, ML ±2.6, DV −4.5/−5.5). Animals for *ex vivo* AAV testing were used from 3 months and up to 5 months after cell transplantation.

### 4.9 Rat brain cryosectioning and immunohistochemistry

Rats were transcardially perfused under sodium pentobarbital anesthesia with 0.9% saline solution followed by ice-cold 4% paraformaldehyde (PFA). The brains were postfixed for 24 h in 4% PFA, incubated in 25% sucrose for 48 h, and sectioned using a freezing microtome to a thickness of 30 to 35 μm in a 1:8 series. Immunohistochemistry was performed on free-floating sections after incubation in Tris-EDTA for 30 min at 80°C for antigen retrieval. Sections were kept in blocking solution [0.25% Triton X-100 and 5% donkey serum in potassium-phosphate buffer (K-PBS)] for 1 h prior to incubation with primary antibody (Supplementary Table 5) and diluted in blocking solution overnight at room temperature. For fluorescent labeling, sections were incubated with secondary antibodies (1:200; Jackson ImmunoResearch Laboratories) and DAPI (1:500) diluted in blocking solution for 1 h at room temperature.

### 4.10 Preparation of acute brain slices

Acute brain slices were prepared from female nude athymic rats (Hsd:RH-*Foxn*1^rnu^). Briefly, animals were decapitated after being deeply anesthetized with 5% isoflurane. The brain was rapidly removed and submerged into an oxygenated (carbogen, 5% CO_2_, 95% O_2_) N-methyl-d-glucamine (NMDG)-HEPES artificial cerebrospinal fluid (in mM): 92 NMDG, 2.5 KCl, 1.25 NaH_2_PO_4_, 30 NaHCO_3_, 20 HEPES, 25 glucose, 2 thiourea, 5 Na-ascorbate, 3 Na-pyruvate, 0.5 CaCl_2_·2H_2_O, and 10 MgSO_4_·7H_2_O. pH was titrated to 7.3–7.4 with 7 mL ± 0.2 mL of 37% hydrochloric acid, and osmolarity ranged from 290 to 305 mOsm/kg. Then, the olfactory bulb and cerebellum were removed to create a tissue block from which coronal slices of about 275 μm were obtained using a vibratome (Leica VT1200 S). The slices were then rinsed with oxygenated pre-chilled (4°C) wash buffer for continued cultures. For detailed procedure we refer to Giacomoni et al. (2023).

### 4.11 Organotypic brain slice cultures and *ex vivo* AAV transduction

Brain slices were cultured as described in Giacomoni et al. (2023). In summary, freshly sectioned brain slices were placed, one per well, on membrane inserts (Millipore) in 6-well plates containing BrainPhys medium with B-27 Supplement, L-Glutamine, and Antibiotic-Antimycotic. On the day after sectioning, culture media was removed until under the membrane, and AAV transduction was performed by placing a droplet of 1.5×10^10^ viral particles per capsid variant at the center of the brain slice overnight. The following day, fresh culture media was added to submerge the brain slices, and cultures were maintained by replacing the culture medium every 2 days. Brain slices were cultured for 10 days to enable GFP expression. Subsequently, they were fixed with 4% PFA for 20 min and then washed 3 times with PBS.

### 4.12 Whole sample immunostaining and optical clearing

The following method was applied to 4% PFA fixed samples of glial spheroids and brain slices according to Giacomoni et al. (2023). Briefly, samples were permeabilized overnight in blocking solution (0.3% Triton X-100 (Sigma), 5% donkey serum, and 0.01% sodium azide in PBS) and then incubated in primary antibodies (Supplementary Table 6) diluted in blocking solution. After 48 – 72 h, samples were incubated with fluorophore-conjugated secondary antibodies (1:200; Jackson ImmunoResearch Laboratories) and DAPI (1:500) for 24–48 h. Finally, samples were dehydrated in an ascending series of methanol concentrations for 10 min each, delipidated with dichloromethane-methanol mixture for 1 h followed by two steps of 10 min each in dichloromethane and cleared with ethyl cinnamate. All samples were kept at room temperature throughout the entire procedure.

### 4.13 Microscopy

Bright-field imaging was performed using an Olympus AX microscope, while fluorescent images were captured using a Leica TCS SP8 confocal laser scanning microscope and acquired using the Leica LAS X software (Leica). DAB-stained brain sections and confocal images of glial spheroids were processed using Adobe Photoshop 2020 (Adobe) or ImageJ (NIH). Confocal images of brain slices were flat fielded using the BaSiC algorithm (Peng et al., 2017) and then stitched using the MIST plugin (Chalfoun et al., 2017) in ImageJ (NIH). Any adjustments were applied equally across the entire image and without loss of information.

### 4.14 High-content screening analysis

Immunolabeled and cleared whole brain slices were placed in 24-well plates with flat and clear bottoms for high throughput microscopy (Ibidi) and imaged with an Operetta CLS high-content imaging system (PerkinElmer) at 10x magnification (Air NA 0.3). 19 stacks (mosaic acquisition; 18 μm z-steps) were taken with no overlap to cover the entire volume. Images were analyzed using Harmony software with PhenoLOGIC (PerkinElmer) and filtered using the sliding parabola method to reduce the fluorescent background. DAPI staining was used to label all nuclei and identify positive staining zones. Within these DAPI^+^ regions, the total number of HuNu^+^, GFP^+^ and PDGFRα^+^ cells, alone or in combination, were counted using the building block “select population” based on a fluorescence intensity threshold for antigen-positive cells, which was defined above internal negative control of background fluorescence from antigen-negative cells. The specificity of each AAV capsid variant for human cells was defined as the number of GFP^+^/HuNu^+^ or GFP^+^/HuNu^+^/PDGFRα^+^ cells over total HuNu^+^ cells. The data were extracted per well/plane/field and plotted using Prism 9.3.1 (GraphPad). The analysis considered only fields with 5% or more human cells out of the total DAPI^+^ per field.

### 4.15 Sequencing RNA-derived barcode

Total RNA was isolated from the glial spheroids using RNeasy Micro Kit (Qiagen) according to the manufacturer’s protocol. RNA samples were incubated with DNase I (NEB) to remove DNA contamination. 5 μg RNA was incubated with 1 unit DNase I in 1X DNase I Reaction Buffer to a final volume of 50 μL and incubated at 37°C for 10 min. Subsequently, 0.5 μL of 0.5 M EDTA was added and heat-inactivated at 75°C for 10 min. DNase I-treated RNA was reverse-transcribed to cDNA using Maxima First Strand cDNA Synthesis Kit (Thermo Fisher) according to the manufacturer’s recommendations. 2 μL cDNA was then amplified by PCR using primers with Illumina Nextera overhangs (Supplementary Table 7) amplifying the viral barcode. The PCR program was one cycle of 30s at 98°C, 30 cycles of 10s each at 98°C, 59°C and 72°C 35 cycles of 5 s at 98°C, 15 s at 65°C and 30s at 72°C followed by one cycle of 5 min at 72°C. The PCR products containing the barcodes were run on an agarose gel. A clean band of the expected 432 bp-size was extracted from the gel extraction kit GeneJET K0692 (Thermo Fisher). Subsequently, a Nextera XT Index PCR using Nextera XT Index Kit (Illumina) was performed. The PCR program consisted of one cycle of 30s at 98°C, 10 cycles of 10s each at 98°C, 59°C and 72°C 35 cycles of 5 s at 98°C, 15 s at 65°C and 30s at 72°C followed by one cycle of 5 min at 72°C. The PCR products were purified using SPRIselect (Beckman Culter). The purified PCR products were sequenced using Illumina NextSeq.

### 4.16 Data assessment workflow

The same data assessment protocol as developed in Davidsson et al. (2019) was utilized. It is a complete interaction-free workflow implemented using the R statistical package. From these scripts, a number of external broad utility applications (bbmap, Blast, Starcode, bowtie2, samtools, Weblogo 3, and Hammock) were called, and the output returned to R for further analysis. This is publicly available as a Git repository^1^ and as a self-sustained Docker image Bjorklund/aavlib:v0.2 with the additional glial analysis available.^2^

### 4.17 AAV-derived barcode identification

From the glial spheroid samples, the AAV-derived barcodes were identified by targeted sequencing as detailed in Negrini et al. (2020). The barcodes are extracted using the flanking constant sequences and clustered together using the Starcode approach to reduce the PCR and sequencing errors. The barcodes are then mapped back to the respective fragments and their origin within the selected proteins utilizing the look-up table from the AAV plasmid. In the last step, the efficacy of transduction was then quantified and mapped with the identification of the most efficient candidates. In parallel, the barcode count and peptide aa sequence were fed into the Hammock tool, and consensus motifs were visualized using Weblogo 3.

### 4.18 Generation of MNM027-MNM030 and MNM031-MNM034

AAV2-derived MNM027-MNM030 was cloned as previously described (Davidsson et al., 2019). The scAAV2 plasmid was digested with NheI (Thermo) and oligos (IDT) containing the peptide sequence, and 22 nucleotide backbone overhang was cloned using HiFi (NEB) according to the manufacturer’s protocol. AAV9-derived MNM031-MNM034 was cloned in multiple steps. First, a peptide stuffer sequence flanked with two recognition sites for AarI was cloned into the AAV9 Cap gene surrounding position 588. This was achieved by a four-fragment HiFi (NEB) assembly and generated an MMTV-AAV2Rep-AAV9Cap (with stuffer at position 588)-ITR-CMV-GFP-pA-ITR. Second, the ITR-CMV-GFP-pA cassette was removed using two NdeI (Thermo) restriction sites and T4 (NEB) ligation. Third, the plasmid [now MMTV-AAV2Rep-AAV9Cap(with stuffer at position 588)]-ITR was digested with AarI (Thermo) overnight to remove the stuffer sequence. HiFi (NEB) assembly with an oligo (IDT) containing the peptide sequence and 25 nucleotides backbone overhang and the AarI digested plasmid was carried out per the manufacturer’s instructions. The resulting capsids were sequenced with Eurofins and Primordium to validate correct insertion.

### 4.19 Statistical analysis

All data are expressed as mean ± standard error of the mean (SEM). All data points have been visualized in the graphs, where each data point represents a biological replicate. Statistical analyses were performed using Prism 9.4.1 (GraphPad). One-way ANOVA non-parametric Kruskal-Wallis test and uncorrected Dunn’s multiple comparisons test or non-parametric multiple Mann–Whitney tests were used as specified in the figure legends. ∗*p* ≤ 0.05, ∗∗*p* ≤ 0.01 and ∗∗∗ *p* ≤ 0.001 are shown; *p* > 0.05 is not shown.

## Data availability statement

The datasets presented in this study can be found in online repositories. The names of the repository/repositories and accession number(s) can be found in the article/Supplementary material.

## Ethics statement

Ethical approval was not required for the studies on human stem cells in accordance with the local legislation and institutional requirements because only commercially available established cell lines were used. The animal study was approved by local ethical committee at Lund University, as well as the Swedish Department of Agriculture (Jordbruksverket). The study was conducted in accordance with the local legislation and institutional requirements.

## Author contributions

JG: Conceptualization, Data curation, Formal analysis, Investigation, Methodology, Project administration, Validation, Visualization, Writing – original draft, Writing – review & editing. MÅ: Data curation, Formal analysis, Investigation, Methodology, Project administration, Validation, Writing – original draft, Writing – review & editing. MH: Data curation, Investigation, Methodology, Project administration, Validation, Visualization, Writing – original draft, Writing – review & editing. AF: Supervision, Writing – review & editing. JK: Methodology, Supervision, Writing – review & editing. MD: Investigation, Project administration, Writing – review & editing. MP: Conceptualization, Funding acquisition, Project administration, Resources, Supervision, Writing – original draft, Writing – review & editing. TB: Conceptualization, Data curation, Formal analysis, Funding acquisition, Project administration, Resources, Supervision, Validation, Visualization, Writing – original draft, Writing – review & editing.

## Footnotes

^1^https://bitbucket.org/MNM-LU/aav-library

^2^https://github.com/MNM-LU/BRAVE-gliaSpheres
