# Supplementary material for: Identification and validation of novel engineered AAV capsid variants targeting human glia
Source: Front Neurosci. 2024 Aug 13;18:1435212. doi: 10.3389/fnins.2024.1435212 (PMC11348808; doi:10.3389/fnins.2024.1435212)
Supplement: Supplementary file 1 [file Data_Sheet_1.pdf]

Supplementary Material

1 Supplementary Figures and Tables

1.1 Supplementary Figures

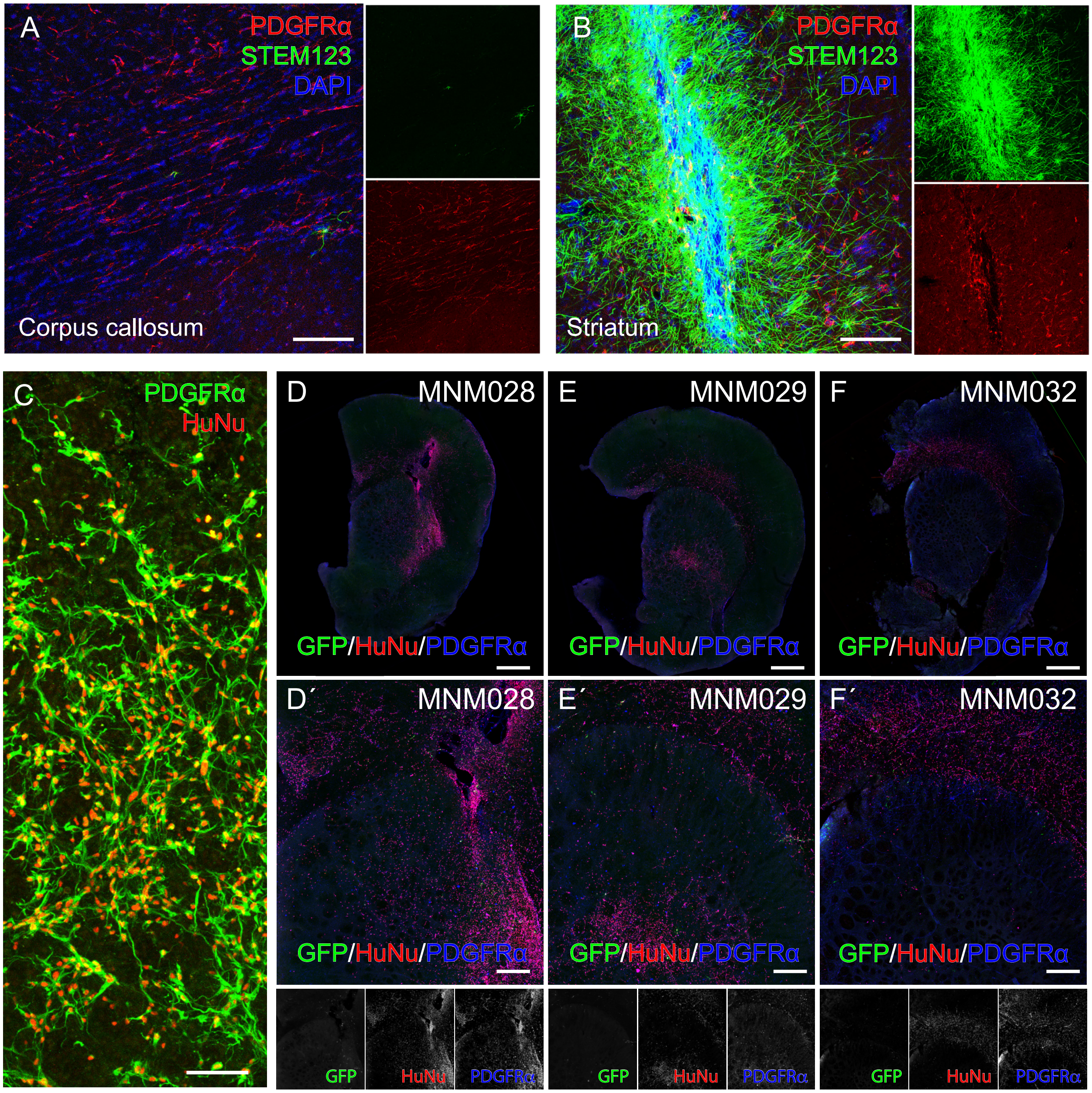

Supplementary Figure 1. (A, B) Maintained expression of GPC identity in cells three months post-transplantation into the rat striatum as visualized using staining for PDGFRα and STEM123 on rat brain cryosections. (C) Confocal immunofluorescence image of an optically cleared brain slice that was not subjected to transduction, revealing the presence of transplanted human cells after ten days of *ex vivo* culture. The cells were identified using stainings for HuNu and PDGFRα. (D-F) Confocal

immunofluorescence pictures of optically cleared brain slices showing minimal to no GFP expression after transduction using MNM028, MNM029 and MNM032, with magnifications (D´-F´). Human glial cells were identified using stainings for HuNu and PDGFRα. Maximum intensity projection of full 275 µm coronal slices.

Data information: scale bars in (A, B, C) 100 µm, in (D-F) 1 mm and in (D´-F´) 500 µm.

1.2 Supplementary Tables

Cell Batch ID	Days in culture	$CD140^+/CD44^-$	$CD140^+/CD44^+$	$CD140^-/CD44^+$	$SSEA4^+/CD133^-$	$SSEA4^+/CD133^+$	$SSEA4^-/CD133^+$
J4	239	47.7%	3.2%	0.5%	0.3%	0.1%	18.8%
LU7	271	62.6%	6.5%	0.3%	0.1%	0.2%	34.6%
LU19	262	44.9%	11.2%	0.5%	1.6%	1.1%	19.6%
J1	219	47.8%	1.4%	0.1%	0.1%	0.0%	21.3%
LU19	263	44.5%	11.9%	0.3%	2.6%	1.2%	13.6%
J4	240	56.5%	2.2%	0.3%	0.9%	0.1%	19.0%
LU7	272	75.6%	5.1%	0.1%	0.2%	0.1%	30.6%
LU13	239	34.5%	27.0%	1.0%	1.0%	0.7%	24.0%
LU28	256	53.5%	3.3%	0.3%	0.4%	0.1%	20.2%
J1	359	34.9%	59.5%	0.3%	0.5%	1.0%	68.0%
LU13	293	59.4%	23.7%	0.2%	0.9%	0.4%	18.1%

Supplementary Table 1. List of hGPCs that were used for the *in vitro* validation of *de novo* generated AAV capsid variants following flow-cytometry analysis at two weeks post-thawing (corresponding to D(-5)). Related to Figure 2M.

	$CD140^+$		$GFP^+/CD140^+$	
Capsid variant	Mean (%)	SEM (±)	Mean (%)	SEM (±)
AAV2 null	58.4	13.0	77.7	9.3
AAV2	38.1	2.9	63.9	6.6
MNM027	56.0	8.0	71.6	6.0
MNM030	51.7	7.4	63.0	6.7
AAV9	56.3	4.6	82.2	2.5
MNM031	67.0	5.4	80.6	3.9
MNM034	51.8	9.0	73.8	5.9

Supplementary Table 2. Percentage of cells analyzed using flow-cytometry from glial spheroids, ten days post-transduction with MNM capsid variants and reference wild-type or null AAVs (biological replicates AAV2 null n = 3; AAV2 n = 4; MNM027 n = 6; MNM030 n = 6; AAV9 n = 9; MNM031 n = 5; MNM034 n= 5). Related to Figures 2P-S.

Cell Batch ID	Days in culture	$CD140^+/CD44^-$	$CD140^+/CD44^+$	$CD140^-/CD44^+$	$SSEA4^+/CD133^-$	$SSEA4^+/CD133^+$	$SSEA4^-/CD133^+$
LU28	245	52.8%	11.5%	1.0%	0.5%	0.6%	41.0%
J4	241	52.5%	11.7%	1.0%	0.9%	0.8%	33.9%
LU28	244	62.6%	11.0%	0.8%	0.3%	0.6%	45.9%

Supplementary Table 3. List of hGPCs batches transplanted into the rat brain following flow-cytometry analysis at two weeks post-thawing. Related to Figure 3.

	$HuNu^+$		$HuNu^+/GFP^+/PDGFR\alpha^+$	
	Mean (%)	SEM (±)	Mean (%)	SEM (±)
AAV2	9.0	0.9	18.0	3.1
MNM027	10.6	1.7	39.8	4.6
MNM030	11.8	1.3	14.4	1.0
AAV9	11.1	1.5	5.9	1.0
MNM031	11.0	1.6	20.9	3.4
MNM034	9.0	1.6	22.8	4.6

Supplementary Table 4. Quantification of the percentage of immunostained $HuNu^+$ cells in optically cleared brain slices, assessed through Operetta CLS, ten days post-transduction with MNM capsid variants and wild-type AAVs (biological replicates AAV2 n = 5; MNM027 n = 8; MNM030 n = 5; AAV9 n = 5; MNM031 n = 6; MNM034 n= 5). Related to Figures 4A,D,E,H.

Antigen	Species	Company (cat. no.)	Dilution
HuNu	Mouse	Millipore (MAB 1281)	1:200
PDGFRα	Rabbit	Cell Signaling Technology (5241S)	1:300
STEM123/hGFAP	Mouse	Takara (Y40420)	1:500

Supplementary Table 5. List of primary antibodies used for immunostaining of rat brain cryosections.

Antigen	Species	Company (cat. no.)	Dilution
GFP	Chicken	Abcam (Ab13970)	1:2000
GFAP	Mouse	Biolegend (SMI 21)	1:500
GFAP	Chicken	Abcam (Ab5541)	1:1000
HuNu	Mouse	Millipore (MAB 1281)	1:200
PDGFRα	Goat	R&D (307na)	1:300
PDGFRα	Rabbit	Cell Signaling Technology (5241S)	1:300
STEM121/hCytoplasm	Mouse	Takara (Y40410)	1:500
STEM123/hGFAP	Mouse	Takara (Y40420)	1:500

Supplementary Table 6. List of primary antibodies used for immunostaining of glial spheroids and *ex vivo* cultured brain slices.

Forward
GTCTCGTGGGCTCGGAGATGTGTATAAGAGACAGGGCATCGACTTCAAGGAGG
Reverse
TCGTCGGCAGCGTCAGATGTGTATAAGAGACAGACGCATCGAGATCGCAGGTGAG

Supplementary Table 7. qPCR primers.
